# RBC Storage Lesion Studies in Humans and Experimental Models of Shock

**DOI:** 10.3390/app10051838

**Published:** 2020-03-07

**Authors:** Willard N. Applefeld, Jeffrey Wang, Steven B. Solomon, Junfeng Sun, Harvey G. Klein, Charles Natanson

**Affiliations:** 1 Critical Care Medicine Department, National Institutes of Health, Bethesda, MD 20892-1662, USA; 2 Department of Transfusion Medicine, National Institutes of Health, Bethesda, MD 20892-1184, USA

**Keywords:** blood transfusion, sepsis, cell free hemoglobin, iron, haptoglobin, shock, pneumonia

## Abstract

The finding of toxicity in a meta-analysis of observational clinical studies of transfused longer stored red blood cells (RBC) and ethical issues surrounding aging blood for human studies prompted us to develop an experimental model of RBC transfusion. Transfusing older RBCs during canine pneumonia increased mortality rates. Toxicity was associated with in vivo hemolysis with release of cell-free hemoglobin (CFH) and iron. CFH can scavenge nitric oxide, causing vasoconstriction and endothelial injury. Iron, an essential bacterial nutrient, can worsen infections. This toxicity was seen at commonly transfused blood volumes (2 units) and was altered by the severity of pneumonia. Washing longer-stored RBCs mitigated these detrimental effects, but washing fresh RBCs actually increased them. In contrast to septic shock, transfused longer stored RBCs proved beneficial in hemorrhagic shock by decreasing reperfusion injury. Intravenous iron was equivalent in toxicity to transfusion of longer stored RBCs and both should be avoided during infection. Storage of longer-stored RBCs at 2 °C instead of higher standard temperatures (4–6 °C) minimized the release of CFH and iron. Haptoglobin, a plasma protein that binds CFH and increases its clearance, minimizes the toxic effects of longer-stored RBCs during infection and is a biologically plausible novel approach to treat septic shock.

## 1. Background

We update here a previous review presenting our most recent red blood cell (RBC) storage lesion studies undertaken in a canine transfusion model [1]. While there have been many insightful investigations and reviews published describing the storage lesion that RBCs accumulate as they age in cold storage [2–5], this article will focus on the insights gained by translational research undertaken by our lab over a decade in a large animal model of shock on the effects of blood transfusion during states of critical illness. These translational animal studies and clinical meta-analytical techniques have given us valuable insights into the pathophysiology of RBC transfusion. RBCs undergo extensive physical and metabolic changes while in cold storage that constitute “storage lesion(s)” [6,7]. Early investigators settled on in vivo RBC recovery and survival at the conclusion of the allowable storage period of 42 days as the key measures of quality. Neither of these surrogate measures of RBC quality has been correlated with patient outcomes. The US Food and Drug Administration (FDA) licenses laboratories that store and distribute RBCs, containers and storage solutions. To date, for licensing, the FDA still relies primarily on these two surrogate measures of efficacy and safety: 24-h survival of >75% of radiochromium-labelled RBCs, and hemolysis of <1% in the storage bag at the end of storage. We sought to determine if transfusion of longer-stored RBCs altered outcomes in critically ill subjects.

We reasoned that clinical trials of transfusing the longest vs. shortest stored RBCs to patients in specific clinical states would pose logistical and ethical problems so we focused on adapting canine models of critical illness (hemorrhagic and septic shock). To minimize unintentional bias, we used mortality as an end point to the RBC transfusion setting. We believe such a model was capable of elucidating any potential risks of transfusing longer-stored RBCs, and if such a risk existed, would allow us to define potential biomarkers of that risk in stored RBCs, explore the mechanism(s) of injury, and test potential interventions.

## 2. Meta-Analysis of Clinical Studies

**Figure 1.**
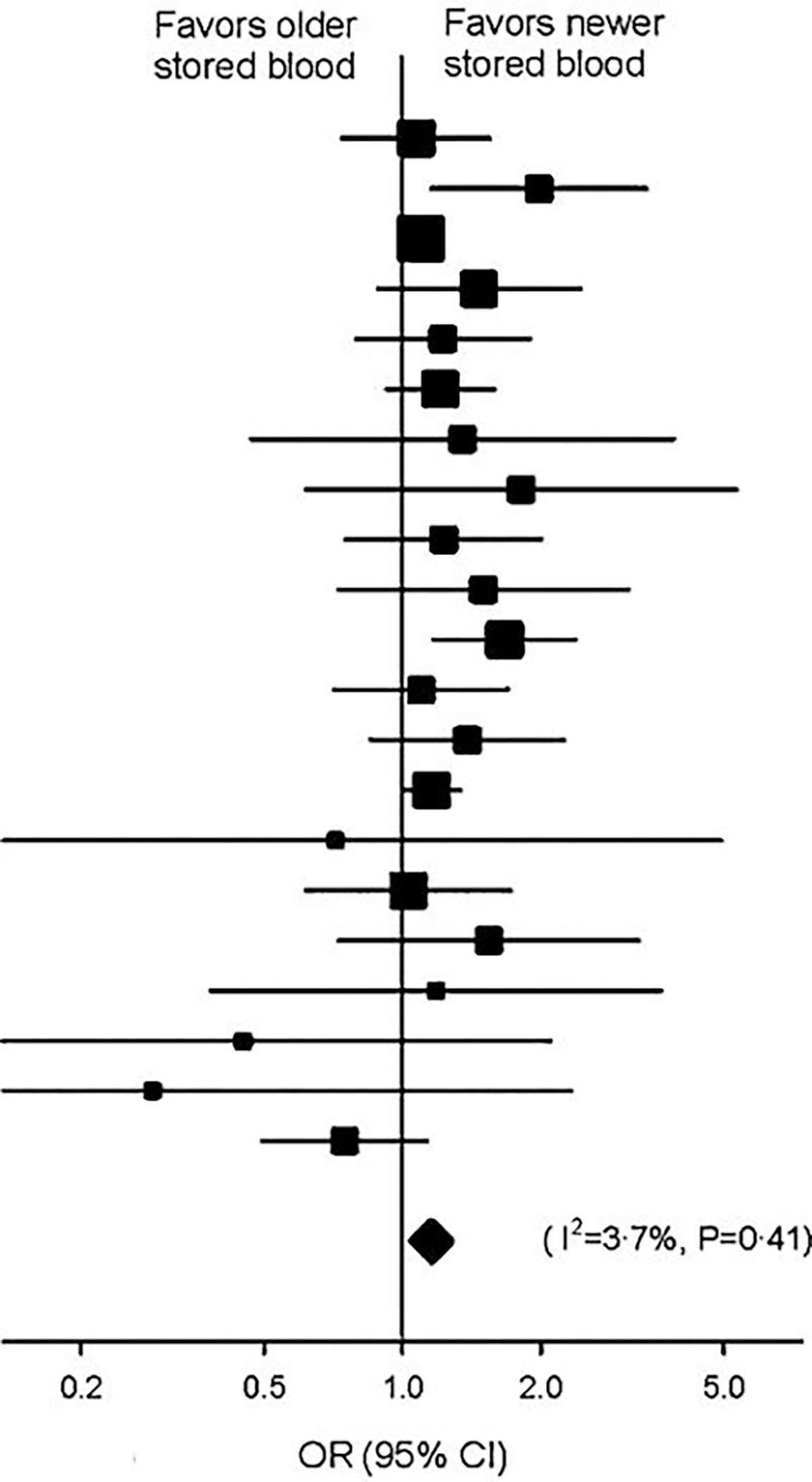
Meta-analysis of the effects of transfusion of old vs. new blood on mortality. The data marker size is proportional to the inverse variance of each point estimate. Meta-analysis of 21 studies revealed a highly statistically significant difference in mortality favoring transfusion of new blood vs. old blood with respect to mortality [odds ratio 1.16, 95% confidence interval (CI) 1.07–1.24, *p* = 0.0001]. From: Wang, D.; Sun, J.; Solomon, S.B.; Klein, H.G.; Natanson, C. Transfusion of older stored blood and risk of death: a meta-analysis. Transfusion 2012, 52, 1184–1195, doi:10.1111/j.1537-2995.2011.03466.x.

We first undertook a review of the scientific literature and undertook a summary of all studies published in English that examined the age of the RBCs transfused and included mortality as a defined outcome. In our meta-analysis of the 21 such studies that met the pre-determined criteria, there was a highly statistically significant difference in mortality comparing the longest stored and freshest conventionally transfused units [odds ratio (OR) 1.16, 95% confidence interval (CI) 1.07–1.24, *p* = 0.0001] favoring transfusion of fresher cells [8] (Figure 1). We did a sensitivity analyses of different important subgroups [studies in different decades, surgery studies, non-surgery studies, adult intensive-care unit (ICU) studies, pediatric studies] within our meta-analysis all yielded consistent findings, further supporting the hypothesis that aging and refrigerating RBC in modern buffer and glucose solutions results in a storage lesion which decreases the safety of transfusion.

## 3. Development of an Animal Model

The preclinical model was designed to study the transfusion of blood at extremes of both storage age (very fresh vs. very long stored) and volume of blood transfused (70% exchange transfusion) in critically ill canines. We reasoned that if there were no differences in mortality under such extreme conditions then it would be fruitless to pursue further studies of RBC storage lesion(s). To accomplish this, we first instilled a known number of colony forming units (CFUs) into the lungs of purpose-bred beagles to produce a 30% to 50% mortality. Then, between hours 4–16 after intrapulmonary bacterial challenge, we exchanged 70% of the canine’s native blood with either fresh vs. old RBCs. This exchange transfusion (80 mL/kg given in 4 divided doses) was performed with fresh (5–7 days of storage) or longer stored (35–42 days of storage) packed RBC units using commercially prepared, universal donor, leukoreduced RBCs. Animals were supported with standard clinical care comparable to that patients with septic shock in an intensive care unit might receive consisting of antibiotics, sedation, mechanical ventilation, fluids, and titrated vasopressor therapy. Animals were treated identically except for the length of time RBC units were stored prior to transfusion. After 96 h, any animal still alive was considered a survivor and sacrificed for necropsy.

## 4. Effects of Longer-Stored versus Fresher Blood Transfusions

**Figure 2.**
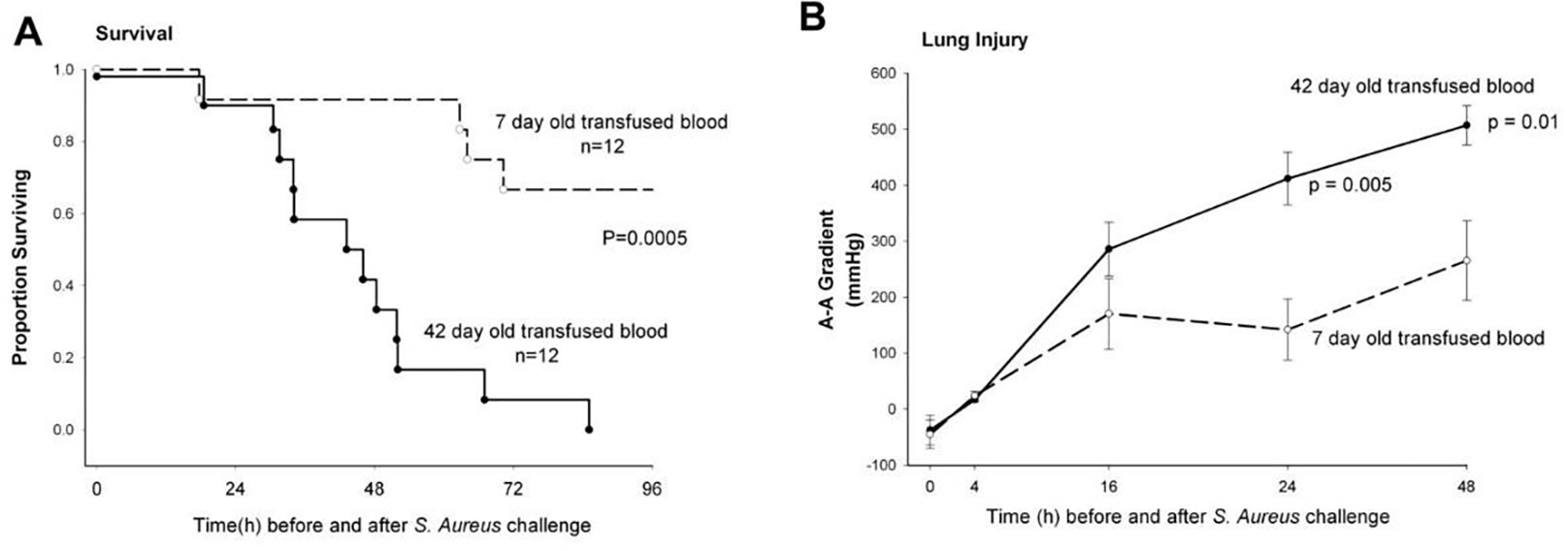
Kaplan–Meier survival curves and lung injury plots for canines receiving longer stored vs. fresher red blood cells (RBCs). (**A**) Proportion of animals surviving over the study period of 96 h comparing canines inoculated with *S. aureus* intrabronchially and subsequently exchange transfused with RBCs stored for 42 days (solid circle, solid line) or RBCs stored for 7 days (open circle, dashed line) RBCs. (**B**) Serial makers of lung injury. Arterial-alveolar oxygen gradient increase demonstrates worsening lung injury in the canines with *S. aureus* pneumonia that received longer stored 42-day-old (solid circle, solid line) vs. fresh 7-day-old (open circle, dashed line) RBCs at 24 h (*p* = 0.005) and 48 h (*p* = 0.01) after bacterial inoculation. From: Solomon, S.B.; Wang, D.; Sun, J.; Kanias, T.; Feng, J.; Helms, C.C.; Solomon, M.A.; Alimchandani, M.; Quezado, M.; Gladwin, M.T., et al. Mortality increases after massive exchange transfusion with older stored blood in canines with experimental pneumonia. Blood 2013, 121, 1663–1672, doi:10.1182/blood-2012-10-462945.

When septic canines underwent RBC transfusion with longer-stored RBCs, there was increased mortality (*p* = 0.0005), worsening of lung injury as measured by the arterial-alveolar oxygen gradient (*p* ≤ 0.01) (Figure 2), and more histologically quantifiable lung damage (*p* = 0.03). After transfusion, animals receiving longer-stored RBCs had evidence of increased in vivo hemolysis with higher levels of both free iron and cell-free hemoglobin (CFH) (*p* ≤ 0.03) [9]. These animals also had elevations in both the mean systemic and pulmonary pressures (all *p* ≤ 0.02) which mechanistically fit with the known vasoconstrictive effect of CFH. This vasoconstriction results from CFH scavenging nitric oxide (NO), a vasodilator. In the animals that did not survive, there was extensive pulmonary necrosis associated with the mortality. This represented one of the first randomized blinded large animal trials demonstrating that mortality increases when blood is transfused at the end of the storage period.

## 5. Mechanisms that Increase Risk after Transfusion of Longer-Stored Red Blood Cells (RBCs) during Infection

This data suggests two main pathways underlying the reported adverse effects of longer-stored RBCs during infection. In the case of both canine and human RBCs, when donor cells are obtained for transfusion they are at different stages in the life span of an RBC. As these donor RBCs age during cold storage, the oldest RBCs likely become even more fragile and prone to in vivo hemolysis after transfusion, resulting in increased release of plasma CFH and iron. Both CFH and iron have well described toxicities that can potentially result in worse outcomes in transfusion settings [10–12]. CFH is well known to cause vasoconstriction through its ability to scavenge NO, an endogenous potent vasodilator. This CFH-induced vasoconstriction can cause ischemia and vascular endothelial injury at sites of infection. Iron serves as a critical nutrient for bacterial growth, potentially worsening infection.

Bacteria associated with human infection commonly produce hemolysins, a virulence factor which can lyse RBCs. Haptoglobin is a natural plasma protein that binds and complexes to CFH. These CFH and haptoglobin complexes, once formed, are sequestered in the intravascular space because of size and then bind to specific receptors on macrophages in the liver and are cleared from the circulation. With excess hemolysis caused both by transfusing longer-stored RBCs and bacteria producing hemolysins, the haptoglobin proteins during infection can become saturated and the reticuloendothelial system is unable to perform the normal function of clearing CFH. This results in excess circulating CFH as well as free and protein-bound iron from RBCs, resulting in increased toxicity during infection.

## 6. Role of Severity of Infection on Risks Associated with Transfused Older Stored RBCs

Next we investigated how increasing the bacterial doses and severity of infection would influence the risks associated with the age of stored blood transfused [13]. Animals underwent intra-bronchial challenge with one of four doses of bacteria; 0 (n = 8), 1.0 × 10^9^ (n = 8), 1.25 × 10^9^ (n = 24), or 1.5 × 10^9^ (n = 8) *S. aureus* colony-forming units/kg and then were exchange-transfused with either fresh (7-day-old) or longer stored (42-day-old) canine universal donor RBCs (80 mL/kg in four divided doses). In both the absence of bacteria and in the presence of increasing doses of bacterial challenges, the levels of CFH and iron were similarly significantly higher when longer-stored RBCs were transfused in comparison to fresh RBCs. However, without bacterial challenge, there was no mortality or measurable end organ injury regardless of the length of time RBCs were stored before transfusion. Infection was required for transfused RBC to increase mortality differentially by length of storage. As the dose of bacterial challenge increased, longer-stored RBCs most profoundly and significantly increased mortality with the middle dose of bacteria. This middle dose of bacteria (1.25 × 10^9^ CFUs) caused, with fresh RBC transfusion and infection, a mortality rate of ~30% which increased to 100% when longer stored RBCs were transfused instead. When animals were challenged with a low dose of bacteria (1.0 × 10^9^ CFUs) and fresh blood transfusion, there was 0 % observed mortality rate and with the highest dose bacteria challenge (1.5 × 10^9^ CFUs) and fresh blood transfusion, there was a 100% observed mortality. Longer stored RBCs nominally but not significantly increased mortality with the low and high dose bacteria. This is in part a power issue regarding the number animals studied, but is also consistent with the notion that a nutrient like iron would have less effect on a mild, less severe infection where iron is unlikely to be able to significantly augment the lethality. In contrast, with the high-dose bacteria, the infection becomes so lethal so rapidly that augmenting it further with a bacterial nutritional factor like iron does not result in an observable difference in this model. These data suggest that an established infection is one of the requirements that augment toxicity for transfused older RBCs. It further suggests that transfusion of older RBCs in septic subjects results in an increased risk from infection and that this risk follows a specific infection dose-response relationship consistent with a nutrient like iron worsening the infection.

## 7. Longer-Stored RBCs in Hemorrhagic Shock

After establishing that older stored RBCs did not harm uninfected controls but were associated with increased lung injury and mortality in a canine model of septic shock from *S. aureus* pneumonia, we next sought to determine if longer stored RBCs would produce similar abnormalities during shock and inflammatory injury in the absence of infection. Therefore, 2-year-old purpose-bred beagles (n = 12) underwent controlled hemorrhage to produce shock and 2.5 h afterward were transfused with similar quantities of either RBCs stored for 42 days or RBCs stored for 7 days [14]. Canines transfused with longer stored RBCs had increases in CFH (*p* < 0.0001) and iron (*p* = 0.004) levels and an unexpectedly lower mortality (18% vs. 50%) from the hemorrhagic shock. The increased levels of CFH with longer-stored RBC transfusion were associated with a more favorable hemodynamic response. With hemorrhage-reperfusion, longer-stored transfused RBCs lowered exogenous norepinephrine requirements (*p* < 0.05) and this lowered cardiac outputs (*p* < 0.05). This hemodynamic effect is consistent with the ability of CFH to cause vasoconstriction by scavenging NO without the cardiac stimulatory properties of norepinephrine. By lowering norepinephrine requirements, elevated CFH in this clinical situation decreased cardiac output which resulted in slower reperfusion after resuscitation which is known to lessen reperfusion injury.

These data in various shock states suggest that host factors mainly dictate if harm, benefit, or no effect will result from the increased hemolysis and liberation of CFH and iron associated with transfusion of older RBCs. In our canine model of septic shock, during transfusion of older blood, the transient increase in iron levels and the rapid removal of iron from circulation is associated with increased lung damage and mortality. These preclinical data are consistent with the notion that during septic shock iron derived from hemolyzed transfused older RBCs disappears more rapidly because iron is utilized by bacteria as a nutrient and growth factor, worsening the infection and increasing mortality. During hemorrhagic shock, elevations of in vivo iron levels persist longer after transfusion of old blood than during infection. Furthermore, in hemorrhagic shock, increased levels of iron are not associated with worsened outcomes but the elevations of CFH lower the need for cardiac stimulatory drugs, which is beneficial. In normal controls, the CFH and iron from transfused longer stored RBCs has no harmful effects. Thus, it is the underlying host factors such as infection or hemorrhagic shock, not the presence of increased CFH and iron alone, that appear critical to determine the outcome from longer-stored transfused RBC.

## 8. Effects of Washing Older versus Fresher Stored RBCs Prior to Transfusion

We next conducted a blinded, randomized controlled study of washing RBCs prior to transfusion in our canine model of septic shock [15]. We hypothesized that the stress of washing older units of blood would cause hemolysis and remove older fragile RBCs prior to transfusion and thereby would improve clinical outcomes by preventing increases of CFH and iron. However, washing fresher units would affect the outcome less. Two-year-old purpose-bred beagles (n = 24) with *S. aureus* pneumonia induced septic shock were exchange-transfused with either fresher (7-day-old) or longer stored (42-day-old) universal donor canine blood (80 mL/kg in 4 divided doses) that had been either washed (commercially employed Haemonetics blood cell processor with commonly used washing procedure, Haemonetics Corp., Braintree, MA, USA) or unwashed. Depending on if the blood was fresh or old, washing RBCs before transfusion had a significantly different and opposite effect on canine survival, organ injury, and plasma iron and CFH levels (all *p* < 0.05 for interactions). Washing older units of blood resulted in improved survival rates, hemodynamic profiles, lung injury, cardiac performance, and liver function as well as reduced levels of iron, possibly by lysing and washing away older damaged cells and CFH and iron in the supernatant. In contrast, when fresh blood was washed, these clinical parameters worsened and levels of CFH increased. Our data suggest that the act of washing fresh blood induces membrane damage to the RBC and, in a setting of established infection, washed RBCs are more likely to lyse in vivo and release iron and CFH which results in worsening outcomes. This finding indicates that fresh RBCs should not be washed routinely, especially in the setting of established infection. Together, these findings suggest that in critically ill subjects with infection, transfusion of fresh blood is preferable to older stored cells as it results in less hemolysis, CFH and iron release, and has proved less toxic than transfusion of older blood. Furthermore, if older blood must be transfused during established infection, washing helps by removing older, more fragile cells mitigating the elevations in plasma circulating iron, improving survival, and decreasing multiple end organ injuries.

## 9. Volume, Age and Washing Effect on RBCs

We next examined the effects of transfusing subjects with washed or unwashed RBCs of varying volumes and age. Two-year-old purpose-bred beagles were inoculated intra-bronchially with *S. aureus* and then were transfused with increasing volumes (5–10, 20–40, or 60–80 mL/kg) of either 7- or 42-day-old RBCs (n = 36) or 80 mL/kg of either washed or unwashed RBCs with increasing age of storage (14, 21, 28, or 35 days) (n = 40) [16]. At all transfused volumes studied (5–80 mL/kg), transfusion of 42-day-old units resulted in similar increases in iron, CFH, lung injury, and mortality rates. Transfusion of 80 mL/kg of RBCs stored for an intermediate duration of time (14, 21, 28 and 35 days) resulted in increased CFH and iron in between levels found when the freshest (7-day-old RBCs) and longest stored RBCs (42-day-old RBCs) were transfused. However, washing RBCs of intermediate ages (14–35 days) did not alter the levels of iron and CFH or change mortality rates. Thus, these preclinical data suggest that by contrast with the extremes of storage age, transfusion of washed blood stored for intermediate periods does not alter risks of transfusion. In contrast, washing any volume of RBCs stored for 42 days potentially decreases CFH and iron levels and decreases risks during established infection. In contrast, transfusion of even massive volumes of fresh RBCs stored for 7 days result in minimal increases CFH and iron levels and risks. Looked at another way, it is not until the last week of storage, week six, that RBCs are so fragile that washing them has net beneficial effects by removing a profound number of cells easily hemolyzed by the stress of washing or transfusion. This increased risk could also be alleviated by decreasing the allowable storage duration to only five weeks. At the National Institutes of Health (NIH), we have applied the results of these preclinical data and have reduced the maximum storage duration for all stored RBCs in the Clinical Center Blood Bank to 35 days.

## 10. The Effects of Intravenous Iron versus Blood Transfusion in Sepsis

We next compared the effects of infusion of intravenous (IV) iron to transfusion of blood in our canine model of sepsis [17]. Clinically, intravenous iron is widely utilized in intensive care units and has been marketed as a safer alternative to RBC transfusion [18,19]. We reasoned that if longer stored RBC hemolyzed in vivo and released iron which augmented the growth of the bacterial challenge, a similar effect might result from direct intravenous iron administration. We compared transfusion of fresh RBCs to two forms of commercially available intravenous iron: iron sucrose, which is an older preparation and the most widely used preparation worldwide, and ferumoxytol, a novel preparation utilizing an iron oxide nanoparticle with a polyglucose sorbitol carboxymethylester coating designed to minimize immunological sensitivity. In our canine model of mild anemia and pneumonia, both iron preparations resulted in similar statistically significantly increased mortality and pulmonary toxicity in comparison to transfusion of fresh 7-day-old RBC [17]. These data suggest that in states of established infection both transfusion of old stored RBC and intravenous iron infusion should be used with caution.

## 11. The Effects of Temperature on RBC Storage Lesion

We next examined the influence of storage temperature on RBC viability using ex vivo human and canine RBCs as well as in vivo radiochromium-labeled RBC transfusions in canines [20]. The 1–6 °C range of temperature variation allowed currently during RBC storage has not changed in over 70 years. Since refrigeration is a key regulatory factor in RBC storage, administration, and transport, a critical re-examination of the effect of storage temperature with contemporary RBC storage practices was long overdue. Our studies revealed that the current practice standards for blood storage does not adequately account for an important interaction between storage time and temperatures that may also confound chromium RBC viability testing and prove clinically relevant.

At the upper bounds of the refrigeration temperature range (4–6 °C), we found that canine and human RBCs have lower RBC viability and increased hemolytic by-products released at near the end of their shelf-life (35 days) when compared to blood stored at lower temperatures (closer to 2–3 °C). RBC hemolysis occurs early within the storage bag and continues as it is stored at 4–6 °C and in vivo after transfusion. Our data suggest that at these higher storage temperatures there is more RBC metabolic activity than at the lower temperatures, resulting in increased lactate levels, more acidosis, lower glucose concentrations, and greater stress to RBC, causing more hemolysis when transfused. This continued hemolytic process can result in shorter intervals between transfusions, the need for additional transfusions, and increased iron deposition for chronically transfused patients.

During the chromium-labelling process (which uses a test sample from a larger unit of blood), the washing procedure, combined with the higher temperature storage, causes lysis of a cohort of the most fragile cells, which results in the test sample taken not being representative of RBC unit being transfused [16]. Storage of RBCs at refrigeration temperatures closer to 2 °C may result in a better product for transfusion and more accurate results on chromium RBC viability testing.

## 12. Updated Meta-Analysis of the Effects of Stored Blood in Clinical Trials

Since our investigations began, there has been an increase in the number of clinical studies investigating the effects of length of storage of blood on mortality and several large randomized controlled trials (RCTs) have been completed. Therefore, we updated our prior meta-analysis [21]. In our analysis of the 31 observational studies, we found a significantly increased risk of death (*p* = 0.01, OR 1.13, CI 1.03–1.24) when patients were transfused with longer stored blood, consistent with our original findings. In our updated meta-analysis, we found there were now more than 4000 patients from six RCTs. We did a separate analysis using the data from the six RCTs and found a significantly different overall effect on outcome from the observational studies (*p* = 0.02 for difference between observational and RCTs), with no survival rate differences between older and fresher RBCs (OR: 0.91, 95% CI: 0.77–1.07) in the RCTs. We attribute the different effect on outcome found in the 31 observational studies vs. the six RCTs to differences in the methods used in the two types of study. Observational studies took current transfusion practice and divided it into two groups of longer stored and fresher blood transfusion, with “longer stored” blood being that which is stored for up to 42 days. In contrast, the RCTs compare current practice, which consisted of blood stored for an average of two to three weeks, to transfusion of the freshest available RBCs which were stored for between one to 10 days. Therefore, in the observational studies, the median age for both the fresher and older blood arms was significantly higher compared to those same groups in the six RCTs (*p* = 0.01). Our analysis of RCTs confirms that the transfusion of the freshest RBCs is not superior to standard practice in which RBCs have been stored for intermediate durations of time (two- to three-week-old). Importantly, the RCTs cannot exclude the possibility suggested by the observational studies that older stored units available for transfusion, namely the four- to six-week-old RBCs, increase mortality risk. After the publication of our systematic review, a large randomized controlled trial conducted at six hospitals in four countries randomizing 31,497 subjects to receive either fresher or older RBC was published and produced findings that are consistent with our findings [22].

## 13. A Fork in the Road: Haptoglobin—A Novel Therapeutic Approach to Sequester Iron and Limit Bacterial Growth and Tissue Injury?

We noted in a randomized controlled trial of our lethal large animal experimental *S. aureus* pneumonia model that there were marked elevations in plasma CFH levels during sepsis, even in the absence of blood transfusion [23]. Hemolysis resulting in elevated CFH levels has been documented to occur during human bacterial sepsis, and higher CFH levels are associated with a worse outcome [24]. Over the last half-century, the overwhelming majority of research on improving sepsis outcomes has unsuccessfully focused on inhibiting host inflammatory mediators. Preclinical studies have found that infusing plasma proteins that bind to sites on the CFH molecule improve outcomes [25,26]. This observation suggests that augmenting host clearance of potential toxic elements such as CHF and iron released from RBCs using a plasma protein transfusion therapy could serve as an alternative pathophysiological approach to treating sepsis.

This alternative pathophysiological therapeutic approach to remove CFH and thereby iron to treat sepsis is thought to evolve as follows: virulent bacteria produce hemolysins which disrupt RBCs and result in the release of CFH, heme, and iron into the vascular space [27,28]. During infection, this can result in injury via multiple mechanisms. CFH itself can scavenge NO, producing vasoconstriction and subsequent tissue and endothelial injury. Free iron serves as an essential bacterial nutrient that promotes bacterial growth and potentially worsens the infection. Additionally, the extravasation of heme into the extravascular space may cause oxidative tissue injury and/or increase inflammatory tissue injury by activating specific pro-inflammatory toll-like receptors. Haptoglobin (Hp), a naturally occurring plasma protein, complexes with CFH and forms high-affinity, high molecular weight CFH-Hp complexes that are subsequently cleared by the reticuloendothelial system [21]. By compartmentalizing CFH to the intravascular space, these CFH-Hp complexes could facilitate CFH and thereby heme and iron clearance during bacterial sepsis, minimize CFH-related tissue damage, reduce the availability of iron to promote bacterial growth, and mitigate heme-induced oxidative injury, thereby possibly improving sepsis survival rates.

## 14. Effects of Haptoglobin Concentrate Administration

**Figure 3.**
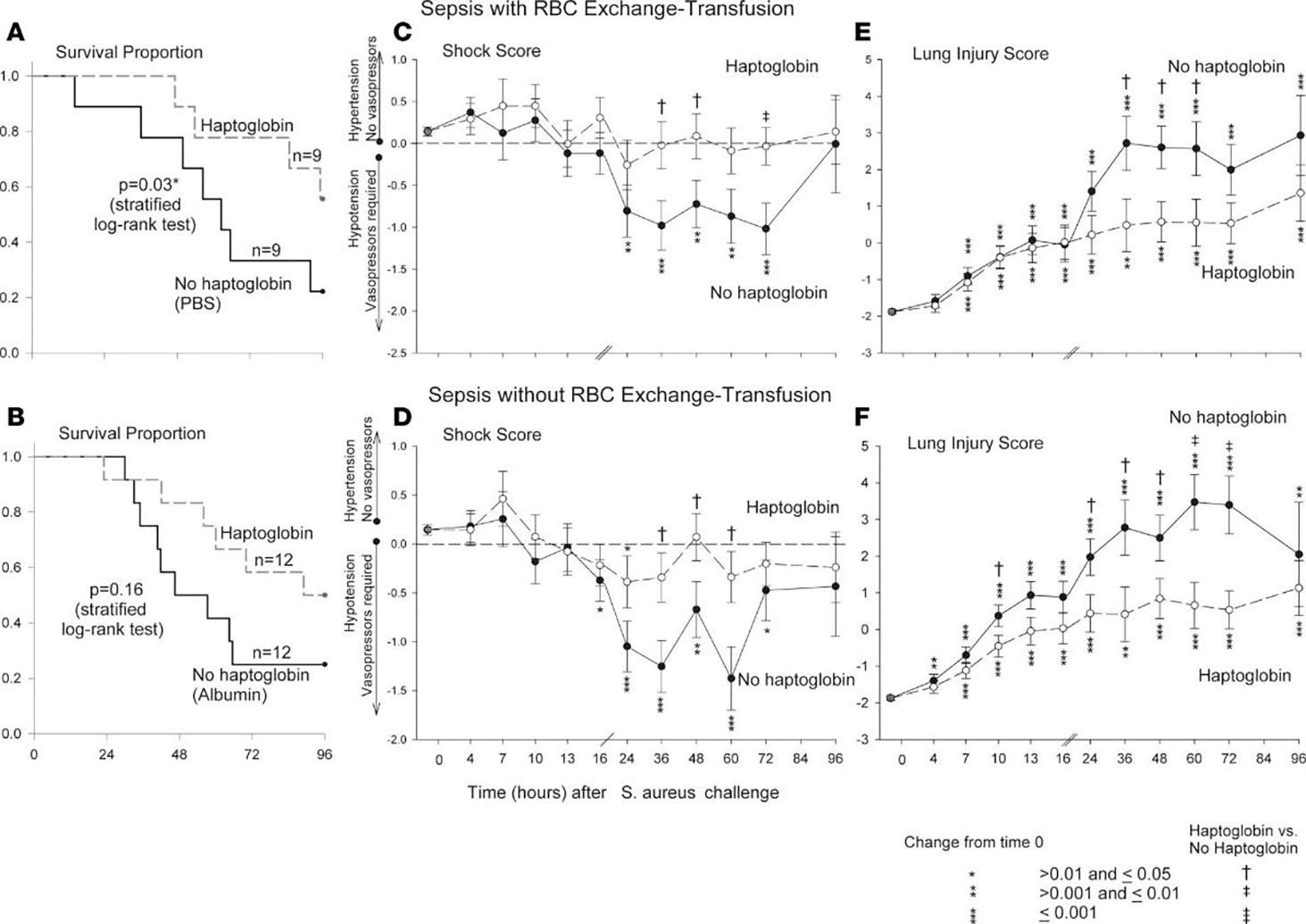
Kaplan–Meier survival curves, hemodynamic assessments, and lung injury plots of septic canines receiving haptoglobin infusion therapy with and without RBC transfusion. (**A**) Proportion of canines surviving after either receiving haptoglobin (dashed line) or no haptoglobin (solid line) infusion after intrabronchial *S. aureus* challenge and subsequent RBC exchange transfusion. (**B**) Kaplan–Meier survival curves of septic animals not undergoing exchange transfusion but receiving haptoglobin (dashed line) or no haptoglobin (solid line) infusions. *P* values denoted by asterisks indicate a statistically significant differences between each panel group using stratified log rank tests. (**C** and **D**) Mean shock scores (+ standard error of the mean (SEM)) at different serial time points. The shock score assesses the amount of vasopressor support (norepinephrine) needed to maintain a given mean arterial pressure (MAP) at the normal level in canines (80 mmHg). The shock score is compared throughout the 96 h study period in canines receiving haptoglobin infusion therapy (closed circle, solid line) and not receiving haptoglobin infusion therapy (open circle, dashed line) in the presence of (**C**) or absence of (**B**) RBC exchange transfusion. The mean baseline values for canines were used to plot a common origin from which changes from baseline were plotted for each study group. Asterisks and their associated *p* values indicate where there are significant changes in each group over time. Crosses and their associated *p* values indicate statistically significant differences between groups receiving haptoglobin infusion and those that did not receive haptoglobin infusion. (**E** and **F**) represent mean (+SEM) lung injury scores (LIS) over serial time points throughout the study. LIS is a global marker of lung damage and represents a composite of mean pulmonary artery pressure, alveolar-arterial oxygen gradient, plateau pressure, oxygen saturation, and respiratory rate. (**E**) demonstrates the LIS over time in canines undergoing exchange transfusion of RBCs receiving (open circle, dashed line) or not receiving (closed circle, solid line) haptoglobin infusions. (**F**) shows the LIS over time in animals not undergoing RBC exchange transfusion but receiving (open circle, dashed line) or not receiving (closed circle, solid line) haptoglobin infusions. Changes in the LIS from baseline are shown for each of the groups plotted from a common origin which was established from the mean value for the canines at baseline. Asterisks and their associated *p* value denote statistically significant changes over time. Crosses and their associated *p* value denote statistically significant differences between the haptoglobin and no haptoglobin group at a given time point. We were able to account for repeated measurements and the pairing of animals within each cycle based on contrasts in linear mixed models which were used to analyze all variables (except survival). From: Remy, K.E.; Cortes-Puch, I.; Solomon, S.B.; Sun, J.; Pockros, B.M.; Feng, J.; Lertora, J.J.; Hantgan, R.R.; Liu, X.; Perlegas, A., et al. Haptoglobin improves shock, lung injury, and survival in canine pneumonia. JCI Insight 2018, 3, doi:10.1172/jci.insight.123013.

Given this potential therapeutic target, we decided to study the effects of commercially available human Hp concentrates in our *S. aureus* pneumonia model of septic shock with and without exchange transfusion of RBCs. To determine if the beneficial effect of Hp therapy was limited only in the setting of blood transfusion, as a control group we also studied haptoglobin infusion in canines with sepsis alone who did not receive transfusion of blood. All septic canines, regardless of whether they received Hp infusion or not, were treated with the same standard clinical interventions employed during human septic shock (e.g., antibiotic, sedation, and titrated cardiopulmonary support). Plasma CFH levels were markedly elevated during sepsis in both groups of animals, those who received blood transfusions and those who did not. When human Hp concentrate was infused over 48 h, regardless of whether the septic animal received blood transfusions or not, there was significant improvement in the metabolic profile as well as reduction in the extent of lung injury, degree of shock, and mortality rates [23] (Figure 3). In both animals receiving blood transfusions and those that did not, improvement was associated with the formation of CFH-Hp complexes, compartmentalization of CFH to the intravascular space with subsequent increased CFH clearance, as well as prevention of the release of free iron and presumably heme.

However, in human septic shock, compared to this preclinical experimental model, there exists a range of lethality and hemolysis with CFH release. This variable amount of hemolysis and CFH release could potentially alter Hp’s beneficial effects. Prior to designing clinical studies in human sepsis, it is important to understand how the CFH-Hp complex kinetics relate to its beneficial effects. Therefore, we performed a second Hp sepsis study in which we addressed two critical questions: (1) in a model of severe and rapidly lethal bacterial septic shock secondary to *S. aureus* pneumonia, would Hp therapy have enough time to provide beneficial effects by reducing the availability of CFH; and (2) in a model of less severe sepsis with lower lethality, could the high levels of CFH produced by transfusing older RBCs result in toxicity from the circulation of large numbers of CFH-Hp complexes [29]?

We found that in both of these circumstances, severe septic shock from *S. aureus* pneumonia as well as increased CFH from transfusion of old blood, the mechanisms of Hp action was disrupted and the previously observed beneficial effects of Hp were no longer observed. In both situations, there was no longer increased clearance of CFH or a reduction of iron levels by Hp and all beneficial effects on outcomes were eliminated [29]. The lack of clearance of the CFH-Hp complexes during severe sepsis suggests that highly lethal infections narrow the therapeutic window for haptoglobin. Extremely elevated levels of CFH after transfusion of longer stored blood were able to saturate the Hp and generate supraphysiologic levels of CFH-Hp complexes. At these levels, transfusion of Hp conferred no beneficial effects on mortality, lung injury, or metabolic parameters in animals receiving Hp and those that did not. We speculate that high levels of CFH-Hp complexes overwhelmed the normal clearance mechanisms, eliminating Hp’s previously observed beneficial effects. This study documented that clearance of CFH and iron are critical to Hp’s beneficial effects during infection. When designing future clinical trials of haptoglobin use in human sepsis, it will be important to understand and account for these factors promoting CFH clearance. If successful, these trials could potentially define the role of transfused haptoglobin concentrates as a therapy for septic shock.

## 15. Conclusions

Designing a clinical trial to deliberately age and then transfuse the oldest blood to determine the risk of the toxicities that develop is ethically problematic. Observational studies support the notion that the longest stored conventionally transfused RBCs increase mortality. Our pre-clinical studies have shown us the pathological changes that blood undergoes over time in cold storage and that its effect is dependent on various host factors including shock states. Our studies suggest that the oldest RBCs, particularly those stored for 7 weeks, can increase hemolysis by releasing iron and CFH, which increases mortality when transfused during infection. Extrapolating clinically, the longest-stored RBCs should not be administered to patients with established infections. At our own institution, we have limited the storage duration of our RBCs to 35 days. Our data also suggest a role for washing blood as it nears five to six weeks of storage; however, the washing process injures fresher RBCs and should not be done with fresh product. We also propose that blood ages differently at different temperatures and is a poorer product with increased hemolysis and iron and CFH release when stored at 4 °C and 6 °C rather than 2 °C. Additionally, we have found in a bacterial model of septic shock that IV iron transfusion is potentially harmful and that until more studies are undertaken, this therapy, similar to blood at the end of the allowed storage period, should be used with caution in patients with established infection. Lastly, the understanding gained from our studies showing longer stored blood having increased hemolysis with release of CFH and iron led to a promising new therapeutic candidate for sepsis treatment—haptoglobin. By compartmentalizing CFH intravascularly, Hp allows CFH to be sequestered and cleared by the reticuloendothelial system, preventing the multitude of negative effects of CFH including NO scavenging, tissue oxidative damage, and iron release with bacterial growth.

## Featured Application:

We have performed a series of studies over the course of a decade with a large animal model of shock and meta-analysis techniques to study transfusion trials. Although clinical trials have established the safety of the current practice of transfusing 18- to 22-day-old stored blood, observational studies have suggested that the oldest units (aged 35–42 days) when transfused have increased risks. These risks are dependent on host factors. Transfused older blood has increased hemolysis with the release of cell free hemoglobin (CFH) and iron. Subjects with infection may be harmed by transfusing iron which promotes bacterial growth. In situations where nitric oxide (NO) is important, such as coronary disease, CFH scavenging of NO may increase risk to patients.
